# Assessment of Anxiety, Depression, Attitude, and Coping Strategies of the Egyptian Population during the COVID-19 Pandemic

**DOI:** 10.3390/jcm10173989

**Published:** 2021-09-03

**Authors:** Ghaydaa A. Shehata, Romany Gabra, Sara Eltellawy, Mohamed Elsayed, Dina Elsayed Gaber, Hatem A. Elshabrawy

**Affiliations:** 1 Department of Neurology and Psychiatry, Assiut University Hospitals, Assiut 71511, Egypt; ghaidaa.abozaid@med.aun.edu.eg; 2 Department of Psychiatry, Faculty of Medicine, Assiut University, Assiut 71511, Egypt; romanyhosny@med.aun.edu.eg; 3 Department of Psychology, Faculty of Arts, Assiut University, Assiut 71511, Egypt; sara_hamdy@art.aun.edu.eg; 4 Department of Psychiatry and Psychotherapy III, University of Ulm, Leimgrubenweg 12-14, 89075 Ulm, Germany; mohamed.elsayed@uni-ulm.de; 5 Department of Neurology, Alexandria University Hospital, Alexandria University, Alexandria 21111, Egypt; Dina.gaber@alexmed.edu.eg; 6 Department of Molecular and Cellular Biology, College of Osteopathic Medicine, Sam Houston State University, Conroe, TX 77304, USA

**Keywords:** coronavirus, SARS-CoV-2, COVID-19, stress, emotions, Egyptian, depression, anxiety, coping strategies, STAI, BDI-II

## Abstract

Background: The COVID-19 pandemic has imposed several challenges on different populations all around the world, with stress being identified as one of the major challenges. This study aims to investigate the impact of COVID-19-induced stress on the prevalence and severity of anxiety and/or depression, factors that predict the development of anxiety and/or depression, and coping strategies in the Egyptian population during the COVID 19 outbreak. Subjects and Methods: This is an observational cross-sectional online study. The questionnaire of our study included five sections: demographic and clinical data, attitude towards COVID-19, the State-Trait Anxiety Inventory (STAI), Beck Depression Inventory-II (BDI-II), and a specifically prepared and standardized Arabic version of a coping strategies scale. The questionnaire was uploaded on 20 May 2020 at 1 p.m. and closed on 7 July 2020 at 8 a.m. Results: The study questionnaire was completed by 283 Egyptians, with mean age 34.81 ± 11.36 years, of which 17% had been infected with COVID-19. The responses showed that 62.9% had moderate anxiety, whereas 12.4% had severe anxiety. Moreover, 13.8% had moderate depression, and 14.1% had severe depression. Our study demonstrated that age, mental status, and being infected with COVID-19 correlated with depression, whereas only age correlated with anxiety. Interestingly, our data showed that anxiety and depression were negatively correlated with some coping strategies during the COVID-19 pandemic. Conclusions: Pandemics, such as the COVID-19 pandemic, imposes stress on individuals, which leads to the development of anxiety and/or depression. Several factors, which could be population-dependent, may help predict the development of anxiety or depression. We show the factors correlated with depression and anxiety during the COVID-19 pandemic in the Egyptian population. Furthermore, certain personal coping strategies during the COVID-19 pandemic are negatively correlated with anxiety and depression. Therefore, our study sheds light on the importance of studying factors in each population that can lead to pandemic-induced psychological complications and those that can relieve such complications.

## 1. Introduction

In December 2019, a COVID-19 outbreak emerged in the city of Wuhan, China. SARS-CoV-2 was soon identified as the novel coronavirus (CoV) causative agent of this outbreak. COVID-19 then spread to all countries in the world, and the disease was declared a pandemic in March 2020 [1]. More than 159 million confirmed cases have been reported worldwide, with 3.4 million deaths as of 10 May 2021. In Egypt, from 3 January 2020 to 10 May 2021, there have been 239,740 confirmed COVID-19 cases and 14,033 deaths [2]. There are several serious impacts of pandemics on different populations, probably due to lack of information about the causative agent, disease progression, and infectious disease control measures [1].

Stress has been reported as a major challenge during the COVID-19 pandemic. Multiple factors that could contribute to stress include the ambiguity and novelty of the COVID-19 pandemic in addition to rapid transmission, high mortality, the unknown actual number of cases, and the unexpected outcome of infection. As a result of stress, levels of anxiety, depression, trauma, suicidal ideation, and panic have elevated during the COVID-19 pandemic. In addition, economic disturbances, community restriction, and uncertainty about the true numbers of COVID-19 cases have contributed to even more stress among the general population.

Reports in the year 2020 have shown that 80% of COVID-19 patients had mild symptoms, high recovery rates, and low mortality rates. However, in comparison to SARS-COV-1 and MERS-CoV combined, SARS-CoV-2 has shown higher transmissibility and mortality. The severe economic sanctions imposed by the governments of several countries, the doubts about the efficacy of personal protective equipment, and fears of shortages in medical supplies can all be reasons contributing to stress among individuals [3,4,5,6,7]. To our knowledge, few studies have investigated the coping strategies of general populations during pandemics. However, personality traits, such as optimism, resilience, and altruism, have previously been shown to have positive effects on reducing psychological stress. In addition, measures such as effective infection control, personal protective measures, clear institutional policies, and protocols have led to stress reduction among various populations [8].

This study aims to evaluate the impact of stress during the COVID-19 outbreak on the prevalence of anxiety and/or depression, predictive factors for the development of anxiety and/or depression, attitude towards COVID-19, and different coping strategies among the Egyptian population in response to the COVID 19 pandemic. Furthermore, we sought to correlate anxiety and depression with different coping strategies.

## 2. Subjects and Methods

This was an internet-based cross-sectional and observational study that was conducted among the Egyptian population. All Egyptian citizens above 18 years were eligible to be included in the study. Each subject was asked to agree to online written informed consent and to complete our questionnaire and submit the response.

### 2.1. Study Participants and Method of Participation

The online semi-structured questionnaire was developed in Arabic by using Google forms, with a consent form appended to it. The link for the questionnaire was sent through various social media platforms (emails, WhatsApp, Facebook, Instagram, Twitter, Reddit). Participants over 18 years old, who agreed to use the online application, were included in this study. Once the link was clicked, the participants were automatically transferred to the information related to the study. Informed consent and demographic details were obtained from all participants. The set of questions of the 15–20 min survey appeared in sequence, which the participants had to answer. The survey questions were designed to assess anxiety and depression in COVID-19 patients and coping strategies for dealing with COVID-19. At the end of the questionnaire, participants had the option to get their results via phone, email, WhatsApp, or other contact methods (optional). Participants also receive some personalized advice according to their results. The data collection was initiated on 20 May 2020 at 1 p.m. and closed on 7 July 2020 at 8 a.m. Data were collected from different governorates in Egypt.

Participants could withdraw from the survey at any moment without providing any justification, and no data was saved. Only data from complete responses to questionnaires were included in our study.

### 2.2. Study Questionnaire

The questionnaire for this study included five sections. All participants were required to understand the meaning of each question and to answer the questions on their own.

#### 2.2.1. Section I

This section included sociodemographic and clinical questions related to age, gender, occupation, education, area of residence, marital status, and chronic illness (if applicable).

#### 2.2.2. Section II

This section included questions about attitudes towards COVID-19, the reaction of the population to COVID-19 patients, and how to deal with others.

#### 2.2.3. Section III. The State-Trait Anxiety Inventory (STAI)

The State-Trait Anxiety Inventory (STAI) is a commonly used measure of trait and state anxiety [9,10]. The standardized Arabic version of the state anxiety scale was used in this study [11] and included 20 items for assessing trait anxiety and 20 items for assessing state anxiety. State anxiety items included “I am tense; I am worried” and “I feel calm; I feel secure”. Trait anxiety items included “I worry too much over something that really doesn’t matter” and “I am content; I am a steady person”. We assessed the state anxiety in the present study. All items were rated on a 4-point scale (e.g., from “almost never” to “almost always”) [12,13]. Higher scores indicate greater anxiety. Internal consistency coefficients for the scale ranged from 0.550 to 0.751, and the value of Cronbach’s alpha coefficient was 0.892.

#### 2.2.4. Section IV. Beck’s Depression Inventory II (BDI-II)

The BDI-II is a 21-item instrument that assesses the presence and severity of unipolar depressive symptoms. A standardized and validated Arabic version of the BDI-II was used [14]. In Beck’s Depression Inventory, individuals rate each statement on a 0 to 3 scale according to how well it describes how they have felt over the past two weeks [15]. A sample item is as follows: “Sadness: 0 = I do not feel sad; 1 = I feel sad much of the time; 2 = I am sad all the time; 3 = I am so sad or unhappy that I can’t stand it”. Total scores were calculated by summing the score of each item. Higher scores indicated greater depression symptoms. In the current study, the value of Cronbach’s alpha coefficient was excellent [14,16,17]. Depression was classified according to the BD-II score into mild, moderate, and severe [14].

#### 2.2.5. Section V. Coping Strategies Scale

The scale was designed specifically for this study. The initial form of the scale included 43 questions to evaluate different coping strategies. The scale was evaluated by five referees to determine the suitability of its items (face validity). The percentage of agreement between the referees ranged from 80–100%. Accordingly, 20 items were deleted, resulting in 23 items in the final form of the scale, which were divided into five dimensions. The responses were graded according to three levels of the Likert scale (applies to me to a great extent = 3, applies to me to some extent = 2, does not apply = 1), and the statements were distributed randomly under different dimensions. The scores are calculated on each subscale. To estimate validity, reliability, and internal consistencies, the scale was applied in its initial form to a survey sample of 188 individuals (pilot study). The validity and reliability of the coping strategies questionnaire have been confirmed by national and international studies, and Cronbach’s alpha coefficient was 0.84.

### 2.3. Statistical Analysis

Data were collected and analyzed using SPSS (Statistical Package for the Social Science, version 20, IBM, Armonk, NY, USA). Continuous data were expressed in the form of mean ± SD or median (range), while nominal data were expressed in the form of frequency (percentage). An independent sample *t*-test was used for continuous variables. A series of correlation coefficients and multiple linear regression analysis were used to examine the predictors for the occurrence of anxiety and depression. Additionally, the relation between different coping strategies and depression or anxiety was studied using Pearson’s correlation. *p* < 0.05 was considered significant.

## 3. Results

### 3.1. Demographic and Clinical Characteristics of the Studied Group

A total of 283 responses from participants who were Egyptians over 18 years old were recorded, mostly from urban areas (90.1%). The mean age of the participants was 34.81 ± 11.36 years; 74.2% were females, and 25.8% were males. About 57.24% of participants exerted high levels of mental effort at work. Our data show that 17% of study participants had COVID-19, whereas 26.1% of participants had chronic illnesses. Our data show that 12.4% of respondents had severe anxiety, and 14.1% had severe depression (according to anxiety rating and Beck’s depression scales; Table 1).

### 3.2. Attitude towards COVID-19

The responses to section II of the study questionnaire revealed the variable attitude of our sample population towards COVID-19. We found that 38.5% of our study population searched for treatment to strengthen their immune system. Moreover, 36.4% had the feeling that they were infected with COVID-19 once a week, whereas 10.6% had this feeling 3 times per week, and 3.9% had this feeling daily.

About one-fourth (*N* = 74; 26.1%) of the sample population had visited a doctor to assure themselves that they were infection-free, while 12% (*N* = 34) underwent a PCR or laboratory test to confirm the absence of infection. Seeking psychiatric consultation occurred in 2.8%, whereas 9.5% of our sample population used anxiolytics. Moreover, 17% of our study participants had a confirmed COVID-19 infection. Unfortunately, all of them reported that they were abused by others, whether by hints, looks, words (62.5%), or by avoidance of direct interaction (37.5%) (Table 2).

### 3.3. Age, Work Nature, and COVID-19 Are Associated with Depression, Whereas Only Age Is Associated with Anxiety

A series of multiple linear regression tests were used to assess the effect of different demographic factors (age, gender, marital status, residence location, and nature of work), clinical factors (chronic illness or having COVID-19), and attitude towards COVID-19 in the development of depression or anxiety among our Egyptian population. We found that age had a significantly negative correlation with depression. However, mental effort at work and COVID-19 disease had significant positive correlations with depression. On the other hand, only age negatively correlated with anxiety (Table 3). These findings indicate that young age, high level of mental effort at work, and COVID-19 disease are significant predictors of depression in our study, whereas young age is the only significant predictor of anxiety in our population.

### 3.4. Anxiety Management Is the Only Coping Strategy That Is Significantly Different between Males and Females

Our newly developed questionnaire was designed to identify the personal coping strategies used by the Egyptian population during COVID-19. Our questionnaire results indicated that anxiety management was the only coping strategy that was more significantly used by females than males (Table 4; *p* = 0.037). The use of personal coping strategies such as ignoring guidelines for disease prevention, following the guidelines of disease prevention, and rational handling of the problem was not significantly different between males and females.

### 3.5. Coping Strategies during COVID-19 Negatively Correlated with Anxiety and Depression

Next, a series of Pearson correlations were performed to correlate coping strategies to the occurrence of depression or anxiety. Anxiety management had a significant positive correlation with anxiety and depression, whereas religious practices had a significant negative correlation with anxiety and depression. However, rational handling of the problem showed a significantly negative correlation only with depression (Table 5). These results indicate that certain coping strategies are effective in reducing the levels of anxiety and/or depression.

## 4. Discussion

The COVID-19 pandemic imposes many challenges that could lead to stress and depression. Different populations react differently to COVID-19-induced challenges. This study reports the prevalence of anxiety and depression due to COVID-19, factors that associate with anxiety and depression, and the coping strategies to deal with the pandemic in a sample of the Egyptian population.

Although our study is a cross-sectional study that does not represent the entire Egyptian population and is limited by the inability to assess incidence or make a causal inference, it provides insight into the reaction of the Egyptian population towards anxiety, depression, and coping strategies to overcome anxiety and depression. Three-quarters of our population had moderate anxiety (62.9%) to severe anxiety (12.4%). This is consistent with a study conducted in Iran, which revealed that approximately one-fifth of Iranians had experienced severe/very severe anxiety [3]. Depression was recorded among half of the studied sample. Most of them had mild (21.9%), moderate (13.8%), and severe depression (14.1%) according to scores of Beck’s Depression Inventory II [18,19]. Another online study reported an increase in anxiety among the Indian population.

Anxiety and depression play a role in immune system dysfunctions and, consequently, increase the risk of viral infection [3]. Moreover, anxiety and depression may trigger public disruptive behaviors due to the attitude of people towards diseased individuals. Panic buying due to anxiety or depression leads to the exhaustion of resources, which could affect daily activities, whereas avoidance behavior causes limited socialization. Furthermore, anxious people can adopt various unwanted lifestyle and dietary modifications under the influence of their emotions.

In our study, 17% of the studied sample had COVID-19, and about 9.9% had direct contact with COVID-19 patients. High levels of anxiety were associated with a negative attitude towards COVID-19 patients. About 12% of our study population performed a laboratory test to check for COVID-19 infection, 26.1% consulted a doctor, 2.8% consulted a psychiatrist, and about 9.5% took medications for anxiety, depression, or sleep disturbance. These attitudes confirm the fear and panic among societies due to COVID-19 and can be attributed to lack of knowledge about the disease, deficiency of protective measures, and mistrusted social media news. To reduce stress, people resort to different strategies, including ignoring the guidelines for the prevention of disease spread, increased religious practices, anxiety management, and rational handling of the problem. All these methods are strategic in dealing with epidemics. In our study, the test to identify coping strategies was designed to be compatible with Egyptian society, with its various challenges. We found that the ability of women to adapt to this pandemic and to relieve emotions was significantly greater than that of men. This can be attributed to the ability of women to endure and adapt to surrounding conditions and the ability of women to better implement protective measures to reduce COVID-19 spread.

Our study showed that younger age, high mental effort at work, and getting infected with COVID-19 were significantly associated with higher levels of depression, whereas younger age was the only factor that was associated with higher levels of anxiety. As a result of increased rates of depression and/or anxiety, more patients sought medical advice and received medications. Next, we sought to determine the effectiveness of coping strategies that were used by our population. Our correlation studies showed that coping strategies such as anxiety management and religious practices were significantly correlated with anxiety and depression. However, rational handling of the problem had a significant negative correlation only with depression. Our results highlight the effectiveness of coping strategies in managing anxiety and/or depression. Therefore, identifying the coping strategies in any population is critical in order to determine which ones will be effective in dealing with stress-related anxiety or depression.

## Figures and Tables

**Table 1:** **Demographic and clinical data of the study population.** Responses to the questionnaire, which included sociodemographic and clinical questions (section I), anxiety and depression assessment questions (sections III and IV, respectively), were recorded. Data are expressed as numbers (*N*) and percentages (%) or mean ± standard deviation (SD).

Variables		Total *N* = 283	
**Age** (mean ± SD)		34.81 ± 11.36	
		*N*	*%*
**Gender**	Male	73	25.8
	Female	210	74.2
**Marital status**	Single	108	38.2
	Married	158	55.8
	Divorced	10	3.5
	Widow	7	2.5
**Nature of Work**	Mental	162	57.2
	Manual	41	14.5
	No work	80	28.3
**Residence Location**	Urban	255	90.1
	Rural	28	9.9
**Chronic Illness**	Yes	74	26.1
	No	209	73.9
**COVID-19**	Yes	48	17
	No	235	83
**Anxiety**	Normal	70	24.7
	Moderate	178	62.9
	Severe	35	12.4
**Depression**	Normal	142	50.2
	Mild	62	21.9
	Moderate	39	13.8
	Severe	40	14.1

**Table 2:** **Population attitude towards the COVID-19 pandemic.** Responses to section II of the study questionnaire, which included questions about the attitudes and reactions of our sample Egyptian population towards COVID-19. Data are expressed as numbers (*N*) and percentages (*%*).

Variables		Total *N* = 283	
		*N*	*%*
**Are you looking for treatments that may help cure or strengthen your immune system?**	Yes	28	9.9
	Scarcely	48	17.0
	Sometimes	126	44.5
	Always	109	38.5
**How many times have you felt you had symptoms like those of COVID-19?**	Did not happen	122	43.1
	A few times (once per week)	103	36.4
	Many times (3 times per week)	30	10.6
	Most of the time (5 times per week)	17	6
	Most of the time (almost every day)	11	3.9
**Have you performed any diagnostic medical tests to check on yourself?**	Did not happen	249	88
	Yes, once a month	18	6.4
	Fortnightly	2	0.7
	Once a week	12	4.2
	More than once every two weeks	2	0.7
**Have you consulted a doctor to check on your health?**	Did not happen	209	73.9
	Once	42	14.8
	More than once	32	11.3
**Did you go to a psychiatrist?**	Yes	8	2.8
	No	275	97.2
**Have you taken any medications for anxiety, depression, or sleep?**	Yes	27	9.5
	No	256	90.5
**The response of population towards you (if you had COVID-19 *N* = 48)**	Some hints by looks or words	30/48	62.5
	Avoidance of direct interaction	18/48	37.5

**Table 3:** **Predictors of depression or anxiety in the Egyptian population.** Linear regression tests were used to determine factors that significantly correlated with depression and anxiety. Linear-regression-dependent (dep.) variables included depression or anxiety, whereas the independent (indep.) variables included age, gender, mental effort at work, marital status, chronic illnesses, and COVID-19 disease.

Dep. Variables	Indep. Variables	Constant	R	R^2^	F	B	SE	Beta	T	*p*-Value	CL 95%
**Depression**	**Age**	20.412	0.205	0.042	12.297	−2.09	0.597	−0.205	−3.51	0.001	{−3.269: −0.918}
	**Mental effort at work**	9.613	0.163	0.027	7.678	4.99	1.799	0.163	2.771	0.006	{1.444: 8.528}
	**Gender**	11.222	0.096	0.009	2.596	2.353	1.461	0.096	1.611	0.108	{−0.522: 5.228}
	**Marital Status**	18.304	0.106	0.011	3.219	−1.751	0.976	−0.106	−1.794	0.074	{−3.672: 0.170}
	**Chronic Illnesses**	13.777	0.05	0.002	0.702	1.228	1.466	0.05	0.838	0.403	{−1.657: 4.113}
	**COVID-19**	1.905	0.201	0.04	11.824	13.05	3.794	0.201	3.439	0.001	{5.578: 20.516}
**Anxiety**	**Age**	51.171	0.117	0.014	3.91	−1.16	0.587	−0.12	−1.976	0.049	{−2.316: −0.005}
	**Mental effort at work**	46.761	0.047	0.002	0.62	1.388	1.766	0.047	0.786	0.433	{−2.088: 4.863}
	**Gender**	45.856	0.060	0.004	1.02	1.43	1.42	0.06	1.01	0.31	{−1.363: 4.226}
	**Marital** **Status**	49.962	0.059	0.004	0.99	−0.95	0.95	−0.06	−0.997	0.32	{−2.816: 0.922}
	**Chronic Illnesses**	46.412	0.065	0.004	1.179	1.541	1.419	0.065	1.086	0.279	{−1.253: 4.334}
	**COVID-19**	48.029	0.030	0.001	0.26	0.24	0.476	0.03	0.51	0.61	{−0.696: 1.179}

**Table 4:** **Coping strategies of males and females in the Egyptian population.** A coping strategies questionnaire was used to identify coping strategies in the population. Respondents were given a score for each strategy based on their answers to the questionnaire. Data are expressed as mean ± SD, and an independent sample *t*-test was used to compare coping strategies between males (*N* = 73) and females (*N* = 210). Asterisk (*) indicates significant difference (*p* = 0.037).

Coping Strategies	Mean Score	Males (*N* = 73) Mean Score	Females (*N* = 210) Mean Score	*p*-Value
**Ignore the Guidelines of Disease Prevention**	9.31 ±2.66	9.68 ± 2.39	9.19 ± 2.73	0.171
**Follow the Guidelines of Disease Prevention**	13.34 ± 3.03	13.41 ± 3.07	13.31 ± 3.02	0.096
**Religious Practices**	12.77 ± 2.24	12.85 ± 2.26	12.75 ± 2.24	0.101
**Anxiety Management**	6.43 ± 1.53	6.11 ± 1.44	6.54 ± 1.55	0.037 *
**Rational Handling of the Problem**	7.25 ± 1.29	7.23 ± 1.31	7.26 ± 1.29	0.891

**Table 5:** **Correlation between depression or anxiety and coping strategies in the Egyptian population.** Pearson correlations were performed to correlate coping strategies to the occurrence of depression or anxiety. Asterisk (*) indicates a significant correlation (*p* < 0.05), whereas (**) indicate significant correlation (*p* < 0.01).

Coping Strategies					
	**Ignore** **the Guidelines of Disease Prevention**	**Follow the Guidelines of Disease Prevention**	**Religious Practices**	**Anxiety Management**	**Rational Handling of the Problem**
**Depression**	R: 0.065 (0.277)	R: −0.077 (0.198)	R: −0.305 (0.0001) **	R: 0.231 (0.0001) **	R: −0.191 (0.001) **
**Anxiety**	R: −0.081 (0.172)	R: 0.051 (0.390)	R: −0.141 (0.017) *	R: 0.277 (0.0001) **	R: −0.075 (0.210)

## Data Availability

Not applicable.
